# Editorial Department

**Published:** 1889-07

**Authors:** 


### EDITORIAL DEPARTMENT.

VETERINARY LEGISLATION.—We beg to inform our readers that the bill to regulate the practice of Veterinary Medicine and Surgery in Pennsylvania, a copy of which will be found on page 95 of the January number of the JOURNAL has passed the Legislature, and has been approved by the Governor.

In the present number we publish copies of the proposed bills to regulate veterinary practice in New Jersey and California, the former of which is almost an exact copy of the Pennsylvania bill, the neat wording of which is noteworthy, while the latter is remarkable for its stringent character. C.

AN ACT TO PROTECT THE TITLE OF VETERINARY SURGEON AND TO REGULATE THE PRACTICE OF VETERINARY MEDICINE AND SURGERY IN NEW JERSEY.

1. *Be it enacted by the Senate and General Assembly of the State of New Jersey,* That every person who shall assume, or use, or cause to be used, any title pertaining to the practice of veterinary medicine or surgery, or any of the branches of veterinary medicine or surgery, shall be a graduate of a legally chartered veterinary college or university, having the power or authority to confer the degree of veterinary surgeon or analogous title, except as provided for in section two; and such practitioner shall be required to register in the book kept for that purpose, in the office of the county clerk of the county in which he resides.

2. *And be it enacted,* That any person who has assumed the title of veterinary surgeon or analogous title, in this State, for the five years preceding the passage of this act, without being entitled to the degree of veterinary surgeon or analogous title, shall be allowed to continue the use of the title; but such person shall appear before the county clerk of the county in which he resides to make affidavit of that fact; he shall then be recorded as an "existing practitioner."

3. *And be it enacted,* That the county clerk shall purchase a book of suitable size, to be known as the "veterinary medical register" of the county, and shall set apart one full page for the registration of each practitioner; and when any practitioner shall die or remove from the county, the county clerk shall make a note of the same, and shall perform such other duties as are required by this act.

4. *And be it enacted,* That every practitioner who shall be admitted to register shall pay to the county clerk the sum of one dollar, which sum shall be compensation in full for registration ; the county clerk shall give a receipt for the same, and such registration shall take place within six months from the passage of this act.

5. *And be it enacted*, That nothing in this act shall be so construed as to prevent any veterinary surgeon (if legally qualified to use the title) from using the title of "veterinary surgeon" or analogous title, in this State; but if such veterinary surgeon opens an office or uses the title for the transaction of business, he shall be deemed "a sojourner," and shall conform to the requirements of this act.

6. *And be it enacted*, That any person who may desire to commence the practice of veterinary surgery or medicine, or any of its branches, in this State, after the passage of this act, and who holds a veterinary diploma, issued, or purporting to have been issued, by any veterinary college or university in this State, another State or foreign country, shall make affidavit before the county clerk that his diploma has been regularly issued by a legally chartered veterinary college or university, after which such person will be allowed to register as provided for in this act.

7. *And be it enacted*, That any person who shall present to a county clerk a veterinary diploma which had been obtained fraudulently, or which is, in whole or in part, a forgery, or shall make affidavit to any false statement, intended to be filed or registered, or shall use the title of veterinary surgeon or analogous title, without conforming to the requirements of this act, or shall otherwise violate or neglect to comply with any of the provisions of this act, shall be deemed guilty of a misdemeanor, and, on conviction, shall be punished for each and every offence by a fine of one hundred dollars, one-half to be paid to the prosecutor, and the other half to be paid to the county, or shall be imprisoned in the county jail in the proper county for a term not exceeding one year, or both or either, at the discretion of the court.

8. *And be it enacted*, That no person shall recover in any court in this State any sum of money whatever for any veterinary, medical or surgical services, unless he shall have complied with the provisions of this act, and is one of the persons authorized by this act to practice as a veterinary surgeon or veterinarian.

9. *And be it enacted*, That in order to secure to the veterinary associations and societies of the State and the State Board of Health a full record of all veterinary physicians and surgeons in this State, it shall be the duty of the county clerk of each county of the State to furnish to all incorporated veterinary associations and societies of the State and to the State Board of Health a list of names of all the veterinary physicians and surgeons who have deposited with him copies of their diplomas, and the name and place of the institution purporting to confer such diploma, and each county clerk shall yearly furnish to the veterinary associations and societies of the State, and to the State Board of Health, a similar list of those veterinary physicians and surgeons hereafter depositing diplomas with him, and shall include in such list also the names of those veterinary practitioners filing affidavits with him as mentioned in the foregoing section of this act ; and each county clerk shall keep in a suitable book an index of the names of all veterinary physicians and surgeons depositing diplomas or filing affidavits in pursuance of the foregoing sections of this act.

10. *And be it enacted,* That this act shall take effect immediately.

---

AN ACT TO REGULATE THE PRACTICE OF VETERINARY MEDICINE AND SURGERY IN THE STATE OF CALIFORNIA.

*The People of the State of California, represented in Senate and Assembly, do enact as follows;*

SECTION 1. That the California State Veterinary Medical Association, a duly incorporated organization, be and is hereby recognized as authority in all questions relating to veterinary science in this State.

§ 2. That it shall be unlawful for any person to practice veterinary medicine and surgery in this State, without having previously obtained a diploma from a college duly authorized to graduate students in veterinary medicine and surgery, or is a member in good standing of the California State Veterinary Medical Association sixty days after the passage of this act, or who has passed a satisfactory examination before the Board of Examiners, as is hereinafter provided.

§ 3. This Board of Examiners, to be known as the State Veterinary Board, consisting of five duly qualified practitioners in veterinary medicine and surgery, is hereby created, whose duty it shall be to carry out the purposes and enforce the provisions of this act. The members of said Board shall be appointed by the California State Veterinary Medical Association. The Board so appointed shall hold their offices for four (4) years. Said Board shall be paid five dollars per day and all necessary expenses while actually engaged in the duties of their office at the meetings of said Board, which shall be held at least once every six months after the appointment of said Board, such meeting to be held alternately in San Francisco and Los Angeles. Said compensation to be paid out of the fees and penalties received under the provisions of this act, and no part of the salary or other expenses of the Board shall ever be paid out of the State Treasury.

§ 4. Said Board of Examiners shall examine all diplomas as to their genuineness. Affidavit shall be made by the holder of a diploma that he is the person mentioned therein, said affidavit being sufficient guarantee that it is genuine. Each applicant not holding a diploma shall submit to a theoretical and practical examination before the Board; said examination to be written or oral, or both, and sufficiently strict to satisfy the Board that the applicant is competent to practice veterinary medicine and surgery. An examination fee of five dollars shall be paid to the Board by the holder of a diploma, and ten dollars by an applicant not holding a diploma, before examination. And in case of failure of approval, said fee shall be forfeited to the Board.

§ 5. All examinations of persons not graduates shall be made directly by the Board, and the certificates given by the Board shall authorize the possessor to practice veterinary medicine and surgery in the State of California. But said examination of ungraduated practitioners must take effect before the thirty-first day of December, 1889. After that date no certificate shall be granted except to persons presenting diplomas from legally chartered veterinary institutions.

§ 6. Upon the approval of the credentials or the examination of an applicant said Board shall grant him a license to practice in this State and shall receive therefor a fee of five dollars, said license to be signed by all members of the Board.

§ 7. Every person qualified, as required by this act, shall, upon receipt of his license to practice, have said license filed in the office of the Clerk of the county in which he resides. Any person removing to another county to practice shall file the license in like manner in the county to which he removes. The holder shall pay to the County Clerk the usual fees for filing. Any failure, neglect, or refusal on the part of any person holding such license to file the same with the County Clerk as above directed, for a period of six months, shall forfeit his license; and no license when once forfeited shall be restored except on the payment to said Board the sum of twenty-five dollars as a penalty for such failure, neglect or refusal.

§ 8. Any person shall be regarded as practicing veterinary medicine and surgery within the meaning of this act who shall profess publicly to be a veterinary surgeon, or appends to his name any initials or title implying qualifications to practice, or who prescribes for or treats sick or injured domestic animals for compensation, either directly or indirectly. But nothing in this act shall be construed to prohibit members of the medical profession from prescribing for domestic animals in cases of emergency, and collecting a fee therefor, nor to prohibit gratuitous services in cases of emergency. And this act shall not apply to commissioned veterinary surgeons in the United States Army.

§ 9. Any person practising veterinary medicine or surgery in this State contrary to the provisions of this act, shall be guilty of a misdemeanor.

§ 10. This Act shall take effect sixty days from and after its passage.

---

APPROPRIATION FOR FREE SCHOLARSHIPS.—The Legislature of Pennsylvania passed an act appropriating the sum of $50.000 payable in two years to the Veterinary Department of the University. The Governor approved the appropriation for one-half the sum $25.000, but vetoed the item for 1890. For this sum the College agrees to educate twelve students each year free of charge. The wealthy citizens of Philadelphia will be called upon to make up the balance of this appropriation, and we trust the appeal will be generously responded to. C.

---

TUBERCULOSIS IN ANIMALS.—Cholera, small-pox and other contagious diseases, when they occur in epidemic form, have been justly considered the most terrible scourge that effects the human race.

There is another disease, equally terrible but more insidious, which cause one-fourth of all deaths occurring in the human family during adult life, tuberculosis. It is likewise a contagious disease, and when it attacks the lungs it is called consumption, and this variety is of especial interest to veterinarians as it is very common among the domestic animals especially cattle.

Doctors Herman M. Biggs, T. Mitchell Prudden, and Henry P. Loomis, pathologists to the New York City Health Department, have recently made a report to that body on the prevention of tuberculosis.

In it they state that the disease is not directly inherited, that it is acquired by the direct transmission of the tubercle bacillus from the sick to the healthy, usually by the means of the dried and pulverized sputum floating as dust in the air, and that it is distinctly preventable.

The measures which they suggest for the prevention of the spread of tuberculosis are :

"First, the security of the public against tuberculous meat "and milk, attained by a system of rigid official inspection of cat-"tle ; second, the dissemination among the people of the knowl-"edge that every tuberculous person may be a source of actual "danger to his associates, if the discharges from the lungs are not "immediately destroyed or rendered harmless ; and, third, the "careful disinfection of rooms and hospital wards that are occu-"pied or have been occupied by phthisical patients."

If these conclusions are accepted as final, the work of the sanitarian, especially in the veterinary profession, will be greatly increased in the endeavors to prevent the transmission of tuberculosis from one human being to another, from animals to man and from man to animals, since the danger to the latter from phthisical attendants must not be forgotten. The importance of quarantine is also manifest. C.

---

INTERNATIONAL CONGRESS OF VETERINARY MEDICINE.—We have received from the committee of organization the following :

The Universal Exhibition of Breeding Horses which will take place in Paris September 1st to 10th, will certainly attract a large number of veterinarians ; the Committee has therefore decided that the International Congress will hold its meetings from Monday the 2nd to Sunday the 8th of September, at the hotel of the Geographical Society, 178 Boulevard Saint Germain.

The subscription remains at 10 francs; it entitles the subscriber to all the publications of the Congress. Subscriptions should be addressed *before the 15th of August* to M. Capon, Trésorier de Comité, 193 rue de l' Université, Paris, France.

During the course of the Congress, the inauguration of the statue of our illustrious master, M. Henri Bouley, will take place at the Alfort School of Veterinary Medicine. The exact date will be announced later.

This Congress will afford an attractive opportunity to meet many of the most illustrious veterinarians of the day, and certainly its meetings will be replete with all the advances recently made in their medicine. The publications of the Congress will be a valuable addition to the library of those who cannot take advantage of the more interesting proceedings. The report of the meeting will be found in the next issue of "The Journal."

